# Exercise Reprograms the Spatial Function of Phosphoglycerate Dehydrogenase of a Pathogenic Nuclear Transcription Factor (PHGDH): A Narrative Review

**DOI:** 10.3390/metabo16030196

**Published:** 2026-03-16

**Authors:** Dong Yang, Wen Guo, Liang Guo

**Affiliations:** 1 College of Physical Education, Hunan Normal University, 529 Lu Shan Nan Road, Changsha 410012, China; yangdong@hunnu.edu.cn; 2 Hunan Provincial Medical Research Center for Intravenous Therapy, 139 Renmin Zhong Road, Changsha 410125, China

**Keywords:** neurodegenerative diseases, PHGDH, exercise

## Abstract

**Background:** Alzheimer’s disease (AD) represents a significant therapeutic challenge, largely attributed to the complex interplay of genetic and non-genetic mechanisms. Among the latter, metabolic dysregulation has emerged as a critical factor influencing disease progression. This study proposes a paradigm shift in our understanding of the role of phosphoglycerate dehydrogenase (PHGDH), a key metabolic enzyme, which, under pathological conditions associated with AD, transitions from a protective role to a pathogenic influence through alterations in its cellular localization and function. **Methods:** To elucidate the impact of exercise on PHGDH dynamics, a narrative review methodology was employed. We conducted comprehensive searches across bibliographic databases, including PubMed, Scopus, and Web of Science, focusing on peer-reviewed articles that detail the relationship between exercise, PHGDH activity, and AD-related neuroinflammation. The review was structured around specific inclusion criteria, which prioritized studies elucidating the mechanisms underlying PHGDH’s dual role in AD pathology and the influence of exercise on this process. **Results:** Our findings reveal that under AD-associated stress, PHGDH translocates to the nucleus, facilitating the activation of pro-inflammatory genes such as IKKα and HMGB1, while simultaneously suppressing autophagy and enhancing amyloid beta (Aβ) deposition. However, exercise induces the release of the myokine irisin, which inhibits PHGDH nuclear translocation through AMPK/PGC-1α signaling pathways. Additionally, peripheral effects of exercise are observed in hepatic Kupffer cells, where exercise attenuates PHGDH activity, leading to reduced systemic IL-1β release and neuroinflammation. **Conclusions:** This study underscores the potential of exercise as a precision intervention in AD management, highlighting its capacity to modulate PHGDH activity and mitigate neuroinflammatory processes. The therapeutic implications of these findings are profound, paving the way for novel diagnostic tools, such as PET probes for assessing PHGDH compartmentalization, and promoting a synergistic approach to “exercise–pharmacotherapy” in the treatment of Alzheimer’s disease. Future research should aim to further delineate the mechanisms by which exercise influences metabolic pathways in the context of neurodegeneration.

## 1. Introduction

### PHGDH: Redefining a Metabolic Enzyme’s Paradigm in Neurodegeneration

Alzheimer’s disease (AD), the most prevalent neurodegenerative disorder, is characterized by progressive cognitive decline, extracellular amyloid-β (Aβ) plaques, and neurofibrillary tangles composed of hyperphosphorylated tau. With the acceleration of global aging, AD currently affects over 55 million people worldwide, and this figure is projected to triple by 2050, imposing an estimated economic burden of USD 1.8 trillion [1]. Despite decades of research, existing therapeutics (e.g., acetylcholinesterase inhibitors and anti-Aβ antibodies) only offer symptomatic relief without halting the progression of the disease. This therapeutic deadlock stems from the multifactorial pathogenesis of AD, where genetic risk factors (e.g., APOE4) account for only 30–50% of sporadic AD (sAD) cases, highlighting the crucial role of non-genetic mechanisms such as metabolic dysregulation and neuroinflammation [2,3].

Phosphoglycerate dehydrogenase (PHGDH), the rate-limiting enzyme in de novo serine biosynthesis, has emerged as a critical node in Alzheimer’s disease (AD) pathogenesis. Longitudinal multi-cohort analyses reveal that PHGDH expression is elevated in both the brain tissue and blood of sAD patients. It correlates with Braak staging and cognitive decline even in asymptomatic individuals, positioning it as a predictive biomarker for early diagnosis [4]. Beyond its canonical metabolic role, PHGDH exhibits a non-canonical function as a transcriptional regulator in astrocytes. Under glucose deprivation, which is a hallmark of AD brains, PHGDH undergoes p38 MAPK-mediated nuclear translocation. There, its helix–helix–turn–helix (HHTH) domain binds the promoters of pro-inflammatory genes (IKKα, HMGB1), acting independently of its enzymatic activity. This binding triggers NF-κB/mTOR activation and suppresses autophagy, accelerating Aβ deposition in 3xTg-AD mice and human brain organoids (BOs) [3]. Crucially, enzyme-dead mutants (PHGDH-ED) retain this pathogenic function, confirming the dissociation of metabolic and transcriptional roles [2].

Therapeutically, the BBB-penetrant small-molecule inhibitor NCT-503 disrupts PHGDH’s DNA-binding capacity by targeting the HHTH domain. In APP-KI mice, NCT-503 reduces hippocampal Aβ plaques by 44–62% and rescues spatial memory deficits without perturbing serine metabolism, underscoring the primacy of transcriptional inhibition over metabolic modulation [2]. These findings caution against serine supplementation—an approach under clinical evaluation—as elevated PHGDH in AD likely augments endogenous serine production, rendering exogenous intake ineffective or potentially detrimental [4].

Epidemiological studies consistently demonstrate that physical activity reduces the incidence of Alzheimer’s disease (AD) by 45% and decelerates cognitive decline in the prodromal stages. Aerobic exercise (AE) has been identified as the most effective modality for enhancing global cognition in elderly AD patients [5,6,7]. Single-nucleus RNA sequencing reveals that exercise restores neuroprotective astrocyte subpopulations (e.g., CDH4^+^ cells) in AD mice. This enhances neurovascular coupling and Aβ phagocytosis, while simultaneously augmenting disease-associated microglial gene expression (e.g., TREM2) to promote amyloid clearance [8,9]. Notably, exercise-induced reactivation of the metabolic gene ATPIF1 promotes neuronal survival and synaptic plasticity, accounting for 64% of transcriptional repair in oligodendrocyte precursor cells [8,9]. Clinically, moderate-intensity continuous training (e.g., 4000 steps/day) increases hippocampal volume by 2%, effectively reversing age-related atrophy and correlating with a 25–50% risk reduction for AD conversion [6,7]. For patients with advanced frailty, pharmacological mimetics targeting exercise-activated pathways (e.g., NCT-503 inhibiting PHGDH transcriptional activity) reduce Aβ plaques by 44–62% and improve spatial memory, offering potential “exercise-in-a-pill” strategies [2].

Consequently, the general objective of this narrative review is to examine the critical intersection of metabolic reprogramming and neurodegenerative disease progression, and to evaluate how exercise interventions serve as emerging therapies for longevity. Specifically, we aim to bridge the gap between understanding the systemic effects of exercise and specific molecular targets in Alzheimer’s disease (AD), moving beyond general observations of neuroprotection to a mechanistic explanation for precision medicine. The specific objectives of this review are as follows: (1) To decode the dual role of phosphoglycerate dehydrogenase (PHGDH) in astrocytes, elucidating the transition from its physiological cytoplasmic metabolic function to its pathogenic role as a nuclear transcription factor under AD-related stress. (2) To assess the mechanisms by which exercise, specifically through the muscle–brain (irisin/AMPK/PGC-1α) and liver–brain (Kupffer cells/IL-1β) axes, reprograms the spatial dynamics of PHGDH to mitigate neuroinflammation. (3) To propose an “exercise–pharmacotherapy” framework that integrates PHGDH targeting with lifestyle interventions as a viable longevity strategy for AD management.

We have specifically narrowed our focus to PHGDH because the current therapeutic landscape for AD has reached a plateau. It often targets late-stage pathological hallmarks (such as amyloid plaques) while ignoring early metabolic instability [4]. PHGDH serves as a critical “metabolic switch” that directly links energetic states to gene expression and immune regulation [4]. Its unique “moonlighting” function—acting as a DNA-binding protein through nuclear translocation—represents a specific therapeutic target for the metabolic-inflammatory cycle implicated in AD [10]. Unlike broad metabolic inhibitors that suppress serine biosynthesis and may compromise neuronal integrity, targeting the HHTH domain of PHGDH allows for the decoupling of its pathogenic transcriptional role from its beneficial metabolic activity [2]. Therefore, understanding how exercise modulates the spatial localization of PHGDH provides a precise mechanistic pathway linking an accessible intervention (exercise) to the molecular underpinnings of the disease, paving the way for personalized treatment regimens that may extend healthspan.

## 2. Methodology

### 2.1. Search Strategy and Data Sources

A comprehensive narrative review methodology was employed to synthesize existing evidence regarding the role of phosphoglycerate dehydrogenase (PHGDH) in Alzheimer’s disease (AD) and the therapeutic potential of exercise interventions. We conducted a systematic literature search on October 2023, utilizing three primary electronic bibliographic databases: PubMed, Scopus, and Web of Science. The search focused on peer-reviewed articles that detailed the relationship between exercise, PHGDH activity, and AD-related neuroinflammation.

### 2.2. Search Strings and Keywords

To ensure a broad capture of relevant literature, we utilized a combination of Medical Subject Headings (MeSH) and free-text terms. The core search string was constructed as follows: (“Alzheimer’s disease” OR “AD” OR “Neurodegeneration” OR “Dementia”) AND (“Phosphoglycerate dehydrogenase” OR “PHGDH” OR “Serine synthesis”) AND (“Exercise” OR “Physical activity” OR “Aerobic exercise” OR “Irisin” OR “FNDC5”) AND (“Neuroinflammation” OR “Astrocytes” OR “Microglia” OR “Nuclear translocation”).

### 2.3. Eligibility Criteria

Inclusion criteria were defined to select high-quality studies directly relevant to the review’s objectives: (1) peer-reviewed articles published in English; (2) studies investigating the canonical metabolic functions and non-canonical transcriptional roles of PHGDH in neurodegeneration; (3) research exploring the mechanisms by which physical exercise modulates metabolic and inflammatory pathways, specifically the AMPK/PGC-1α axis and liver–brain crosstalk; (4) both preclinical (animal and cellular models) and clinical studies were considered. Exclusion criteria included: (1) non-peer-reviewed articles (e.g., editorials, opinion pieces); (2) conference abstracts; (3) studies that did not address PHGDH, exercise mechanisms, or specific neurodegenerative contexts.

### 2.4. Rationale for Article Selection

Articles were selected based on their contribution to elucidating the “dual role” of PHGDH—as both a metabolic enzyme and a nuclear transcription factor—and its regulation by physical activity. Priority was given to studies providing mechanistic evidence on how exercise-induced myokines (e.g., irisin) and hepatokines influence PHGDH compartmentalization and activity, thereby bridging the gap between systemic metabolic health and central nervous system pathology. The selected literature collectively supports the paradigm shift view of exercise as a precision intervention capable of reprogramming the spatial function of PHGDH (Table 1).

## 3. The Metabolic and Transcriptional Duality of PHGDH in Astrocytes: A Bifunctional Regulator in Neurodegenerative Pathogenesis

Phosphoglycerate dehydrogenase (PHGDH), the gatekeeper of the serine biosynthesis pathway, is canonically defined by its catalytic conversion of 3-phosphoglycerate (3-PG) to 3-phosphohydroxypyruvate (3-PHP), a process integral to one-carbon metabolism and nucleotide biosynthesis [10]. This traditional view is now being challenged by the revelation of a non-canonical, moonlighting function for PHGDH as a transcriptional regulator, a role that is especially pronounced in astrocytes—the principal glial population of the central nervous system (CNS) [2]. This functional duality situates PHGDH at the confluence of metabolic reprogramming and epigenetic regulation, positioning it as a pivotal player in the pathogenesis of neurodegenerative diseases, notably Alzheimer’s disease (AD) [10,13]. In the context of AD, astrocytes—long relegated to the status of metabolic support cells for neurons—undergo a pathological reprogramming characterized by aberrant PHGDH expression. Strikingly, the magnitude of this dysregulation scales with Braak staging and parallels cognitive deterioration [13].

### 3.1. Metabolic Functions: Serine Synthesis and Beyond

**Figure 1:**
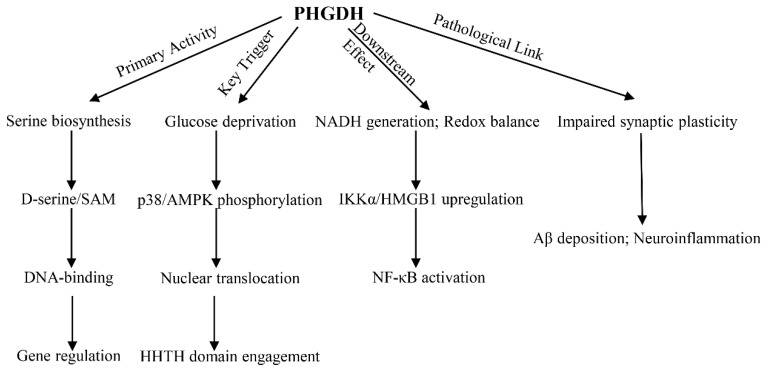
The dual functions of PHGDH in astrocytes. Normal metabolic function: under physiological conditions, PHGDH is primarily located in the cytoplasm, serving as the rate-limiting enzyme in the serine synthesis pathway. It generates D-serine, a co-agonist of NMDA receptors, to maintain synaptic plasticity, while also producing the epigenetic modifier SAM through serine metabolism. Pathological Transcriptional Function: In pathological states such as Alzheimer’s disease, PHGDH is phosphorylated by p38 MAPK and translocates into the nucleus. Its HHTH Broussonetia papyrifera domain acts as a transcription factor, directly activating pro-inflammatory genes such as IKKα and HMGB1. This process inhibits autophagy via the NF-κB pathway, accelerates Aβ deposition, and leads to neurodegeneration.

In astrocytes, phosphoglycerate dehydrogenase (PHGDH) serves as the key regulator of de novo serine synthesis, generating D-serine, an indispensable co-agonist of NMDA receptors for synaptic plasticity (Figure 1). Genetic deletion of PHGDH in murine models depletes D-serine reserves, thus impeding long-term potentiation (LTP) and spatial memory [13]. Beyond its function in neurotransmission, serine derived from PHGDH sustains the folate cycle, which generates S-adenosylmethionine (SAM). SAM acts as the universal methyl donor for reactions that regulate histone modifications (e.g., H3K27me3) and DNA stability in neurons [14].

Under conditions of glucose deprivation—a hallmark of Alzheimer’s disease (AD) brains—PHGDH undergoes dynamic subcellular redistribution. Specifically, p38 MAPK phosphorylates PHGDH at Ser371, triggering its nuclear translocation (Figure 1) [10]. Concurrently, AMPK-mediated phosphorylation at Ser55 reprograms PHGDH’s catalytic activity to utilize alternative substrates like malate. This metabolic shift generates NADH to sustain redox homeostasis [10]. Such metabolic plasticity enables astrocytes to maintain the NAD^+^/NADH balance during energetic stress, a process elegantly visualized using nuclear-targeted SoNar/Frex biosensors [10].

### 3.2. Transcriptional Regulation: The Non-Canonical Paradigm

In astrocytes within the Alzheimer’s disease (AD) brain, PHGDH assumes a novel, non-canonical role in the nucleus, functioning independently of its well-characterized metabolic activity. Using its helix–helix–turn–helix (HHTH) domain, PHGDH acts as a DNA-binding protein, as shown by chromatin immunoprecipitation sequencing (ChIP-seq), which identified its occupancy at promoter regions. This binding directly promotes the expression of key pro-inflammatory genes, IKKα and HMGB1 (Figure 1) [10]. The downstream effects are highly pathogenic: IKKα enhances NF-κB signaling, while HMGB1 impairs the autophagic removal of amyloid-beta (Aβ), establishing a self-perpetuating cycle that increases amyloid deposition [13].

This dual functionality is unequivocally demonstrated by the finding that enzyme-dead PHGDH mutants (e.g., PHGDH-ED) retain their transcriptional capabilities, confirming a clear dissociation between its metabolic and gene-regulatory functions (Figure 1) [10]. The HHTH domain (residues 210–310) is the critical mediator of this activity. Its deletion via mutagenesis prevents PHGDH from activating IKKα and HMGB1, even when the protein is overexpressed to pathological levels [10]. Furthermore, the structural homology of this domain to established transcription factors provides the molecular basis for its ability to engage in sequence-specific DNA recognition, specifically targeting CpG-rich sites near neuroinflammatory gene promoters [10].

### 3.3. Pathogenic Implications in Neurodegenerative Diseases

Elevated RNA and protein levels of PHGDH in astrocytes have been consistently demonstrated in multi-cohort analyses of late-onset Alzheimer’s disease (LOAD). These levels positively correlate with amyloid-beta (Aβ) burden and Braak staging [13]. This relationship is further supported by functional studies in 3xTg-AD mice. In these studies, astrocyte-specific overexpression of PHGDH resulted in a 2.3-fold increase in Aβ plaques. In contrast, PHGDH knockdown led to a substantial reduction of 60–70% in plaque formation [13]. At the mechanistic level, the PHGDH-IKKα-HMGB1 axis contributes to Aβ accumulation by suppressing autophagy and enhancing the activity of β-secretase (BACE1) [13].

The function of nuclear PHGDH extends to the integration of metabolic stress with immune responses. Under conditions of low glycolytic flux, such as the depletion of 3-phosphoglycerate (3-PGA), PHGDH promotes the assembly of a PHGDH-AXIN-HIPK2 complex. This complex facilitates the phosphorylation of p53 at Serine 46, initiating pro-apoptotic pathways in astrocytes under duress [12]. On the other hand, through its role in producing S-adenosylmethionine (SAM), PHGDH fuels the trimethylation of H3K27 (H3K27me3) at the promoters of key neuroprotective genes like BDNF, leading to their transcriptional silencing and a subsequent increase in neuronal vulnerability [13,14].

## 4. Multi-Organ PHGDH Network Remodeling Underlies Systemic Benefits of Exercise Training

### 4.1. Exercise-Induced Irisin Modulates the Brain Metabolic Landscape via AMPK/PGC-1α-Mediated Suppression of PHGDH Nuclear Translocation

Physical exercise emerges as a potent non-pharmacological intervention that consistently demonstrates neuroprotective benefits in both clinical and preclinical studies. Intriguingly, the muscle–brain endocrine axis has been implicated as a critical mediator of exercise-induced neuroprotection, with the myokine irisin serving as a key signal transducer. This review presents a novel hypothesis: Exercise-derived irisin activates the AMPK/PGC-1α axis to suppress the nuclear translocation of phosphoglycerate dehydrogenase (PHGDH), thereby reprogramming brain metabolism and attenuating neurodegenerative pathology [15,16].

Skeletal muscle functions as a dynamic endocrine organ during physical activity, secreting myokines that communicate with distant organs, including the brain. Irisin, a cleaved fragment (∼12 kDa) of the membrane protein fibronectin type III domain-containing protein 5 (FNDC5), is robustly upregulated by exercise via the PGC-1α-dependent pathway in contracting muscle. Upon release into circulation, irisin crosses the blood–brain barrier (BBB) via saturable transport mechanisms, achieving physiologically relevant concentrations in the hippocampus, cortex, and cerebrospinal fluid. Crucially, irisin deficiency exacerbates synaptic loss and cognitive impairment in AD models, whereas its administration rescues neurogenesis deficits [16]. This establishes irisin as a principal exercise-derived mediator of central nervous system (CNS) adaptation.

Irisin initiates a neuroprotective signaling cascade by binding to integrin αV/β5 receptors on the surface of neurons and glial cells. This interaction triggers the activation of AMP-dependent protein kinase (AMPK), a master cellular energy sensor that is phosphorylated at Thr172 in response to irisin-induced shifts in the cellular AMP:ATP ratio. Once active, AMPK directly phosphorylates peroxisome proliferator-activated receptor gamma coactivator 1-alpha (PGC-1α) at Ser538, which enhances its stability as a transcriptional coactivator and promotes its retention within the nucleus [17]. In turn, PGC-1α orchestrates a transcriptional program that favors mitochondrial biogenesis—mediated by NRF1 and TFAM—and bolsters antioxidant defenses through SOD2 and CAT. Notably, activation of the AMPK/PGC-1α axis has demonstrated neuroprotective effects in ischemic stroke models, correlating with improved mitochondrial membrane potential and enhanced ATP generation [17].

Phosphoglycerate dehydrogenase (PHGDH), which catalyzes the rate-limiting step of de novo serine biosynthesis, diverts glycolytic flux toward amino acid production and one-carbon metabolism. Under pathological stress conditions, such as exposure to amyloid-beta (Aβ) oligomers, PHGDH undergoes translocation to the nucleus. Within the nucleus, it locally generates serine to support histone methylation (specifically H3K27me3), leading to the repression of genes essential for neurogenesis. This elevation of nuclear PHGDH correlates with impaired hippocampal neurogenesis and deficits in memory consolidation in models of Alzheimer’s disease (AD). We hypothesize that exercise-induced irisin signaling suppresses this aberrant nuclear localization. To mechanistically validate this axis, we will investigate whether exercise intervention fails to suppress PHGDH nuclear translocation in Fndc5 murine models, thereby exacerbating tau hyperphosphorylation through the amplification of this pathological cascade.

### 4.2. Exercise-Mediated Suppression of Hepatic Macrophage PHGDH Attenuates Neuroinflammation via the Liver–Brain–Immune Axis

Neurodegenerative diseases, including Alzheimer’s disease (AD) and Parkinson’s disease (PD), are characterized by progressive neuronal loss and neuroinflammation driven by microglial activation and cytokine dysregulation (e.g., IL-1β, TNF-α). While central mechanisms have been extensively studied, recent evidence highlights the liver as a critical peripheral regulator of brain immunity via the “liver–brain–immune axis” [12,17]. This axis orchestrates systemic metabolic and immune homeostasis, where hepatic macrophages (Kupffer cells) act as key sentinels. Intriguingly, physical exercise—a non-pharmacological intervention—modulates this axis by suppressing phosphoglycerate dehydrogenase (PHGDH) in hepatic macrophages, thereby reducing IL-1β production and mitigating neuroinflammation [2,17].

PHGDH catalyzes the rate-limiting step in de novo serine synthesis, a pathway linked to inflammatory responses. In aged or metabolically stressed livers, PHGDH is upregulated in Kupffer cells, promoting serine flux toward IL-1β biosynthesis via the NLRP3 inflammasome. Serine-derived metabolites fuel mitochondrial respiration, enhancing ROS generation and caspase-1 activation, which cleaves pro-IL-1β into its active form [2]. Elevated IL-1β enters the systemic circulation, crosses the compromised blood–brain barrier (BBB) in neurodegeneration, and activates microglia, exacerbating Aβ/tau pathology in AD and α-synuclein aggregation in PD [18]. Critically, PHGDH inhibition reduces IL-1β by >40% in murine models, confirming its pro-inflammatory role [2].

**Figure 2:**
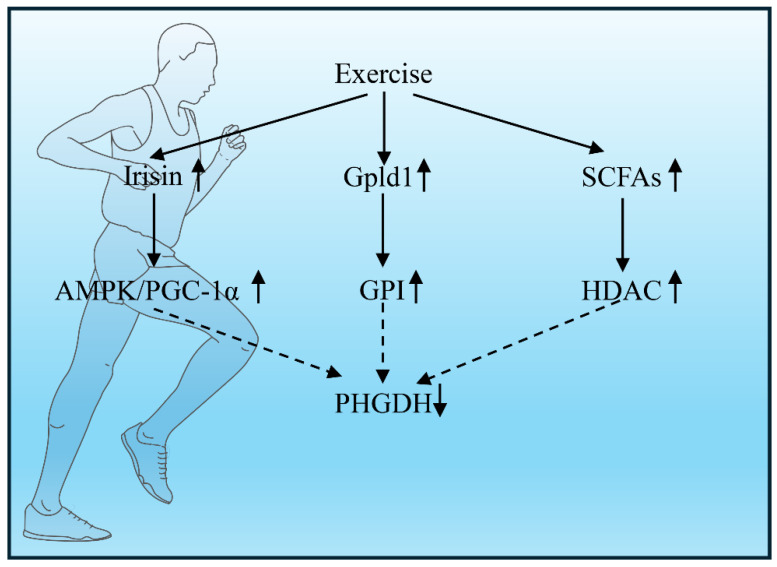
Molecular Pathways Linking Exercise to Hepatic PHGDH-IL-1β Suppression. Central pathway (Intra-brain): Exercise induces the release of the myokine irisin from skeletal muscle. Irisin crosses the blood–brain barrier and activates the AMPK/PGC-1α signaling pathway in the brain (primarily in astrocytes), thereby inhibiting PHGDH nuclear translocation and retaining it in the cytoplasm to perform its beneficial metabolic functions. Peripheral pathway (liver–brain axis): Exercise modulates hepatic immunity by suppressing PHGDH in liver macrophages (Kupffer cells), reducing the systemic release of the pro-inflammatory cytokine IL-1β, which in turn mitigates neuroinflammation in the brain.

Exercise induces multi-organized crosstalk that converges on hepatic PHGDH regulation (Figure 2):

- AMPK/SIRT1 Activation: Exercise initiates a powerful metabolic signal by releasing myokines (IL-6, irisin) from skeletal muscle. These myokines activate hepatic AMPK and SIRT1. These enzymes orchestrate a two-pronged attack on PHGDH: AMPK flags PHGDH for destruction by phosphorylating it at Ser49, while SIRT1 suppresses PHGDH gene transcription by deacetylating NF-κB [12,19].
- Gpld1-Mediated Signaling: The liver counters inflammation through the exercise-induced hepatokine Gpld1. Gpld1 disrupts inflammatory signaling in Kupffer cells by cleaving GPI-anchored proteins, which directly halts PHGDH-driven serine synthesis and blocks IL-1β maturation. This pathway is so crucial that its absence in Gpld1-deficient mice renders the anti-inflammatory effects of exercise completely ineffective [12,19].
- Gut-Liver Axis Modulation: Exercise also reshapes the gut ecosystem, fostering beneficial bacteria such as Bifidobacterium. These microbes generate short-chain fatty acids, particularly butyrate, which act as epigenetic regulators. In Kupffer cells, butyrate inhibits histone deacetylases, effectively silencing the expression of key inflammatory genes, PHGDH and IL1B [12].

## 5. Precision Exercise Intervention: Diagnostic and Therapeutic Innovations Targeting PHGDH

Based on a comprehensive review of the current literature, we have identified several promising avenues for PHGDH-targeted precision exercise interventions that merit further investigation. These approaches are logical extensions of existing research and highlight innovative strategies to bridge the gap between mechanistic understanding and clinical application.

Aim 1: To develop diagnostic tools for PHGDH subcellular dynamics. Current research suggests that quantifying the PHGDH nuclear-to-cytoplasmic ratio (N/C ratio) could serve as a valuable biomarker for early AD detection and patient stratification. The development of PET probes engineered with the HHTH structural domain represents a frontier in molecular imaging that could enable in vivo mapping of PHGDH compartmentalization. Such diagnostic innovations would allow clinicians to identify patients with aberrant PHGDH nuclear translocation before significant cognitive decline occurs, facilitating earlier and more targeted interventions.

Aim 2: To explore synergistic exercise–pharmacotherapy approaches. Preclinical evidence indicates that combining aerobic exercise with PHGDH-targeted pharmacotherapy may yield synergistic benefits. Studies suggest that exercise may modulate blood–brain barrier (BBB) function and transport mechanisms, potentially influencing the CNS bioavailability of compounds like the PHGDH inhibitor NCT-503. However, it is important to note that the effects of exercise on BBB are complex and context-dependent, involving both structural and functional adaptations that may vary with exercise type, intensity, duration, and disease state. Future research should systematically evaluate this potential interaction through well-designed preclinical models that incorporate both exercise interventions and pharmacological agents, with careful assessment of pharmacokinetic and pharmacodynamic profiles.

Aim 3: To optimize combinatorial therapy parameters. Translating these interventions into clinical practice requires establishing evidence-based protocols for combined exercise and pharmacotherapy. Current literature suggests that determining optimal parameters—including dosing regimens for PHGDH inhibitors and exercise prescription variables (intensity, duration, frequency)—is crucial for maximizing therapeutic outcomes. This optimization should be guided by both pre-clinical efficacy data and emerging clinical evidence, taking into account individual patient factors such as disease stage, physical capacity, and metabolic profile.

These proposed directions build upon the mechanistic insights presented throughout this review, offering a framework for translating our understanding of PHGDH biology into practical interventions for AD prevention and treatment.

## 6. Precision Exercise Targeting PHGDH in Neurodegenerative Diseases: Challenges, and Future Experimental Designs

To further explore the therapeutic potential of gene-exercise crosstalk, we will employ AAV-mediated delivery of a constitutively active PHGDH-S553D gene variant, engineered to mimic exercise-induced phosphorylation. This innovative strategy requires a sophisticated experimental framework that incorporates the following: (1) Quantitative evaluation of transduction efficiency using advanced molecular techniques. (2) Longitudinal therapeutic monitoring through multimodal imaging and functional assays. (3) Rigorous safety profiling to assess potential off-target effects.

Through an interdisciplinary collaboration integrating biomedical engineering, molecular neuroscience, and clinical neurology, we anticipate achieving transformative breakthroughs in mitigating the progression of motor neuron degeneration.

A central objective of our future experimental design is to elucidate a fundamental question: how does exercise specifically regulate the HHTH domain of PHGDH without affecting its canonical enzymatic activity? To comprehensively dissect this complex mechanism, we have devised two complementary experimental strategies.

First, we will leverage cutting-edge spatial omics technologies by integrating single-cell sequencing with subcellular proteomics to map the comprehensive landscape of the PHGDH interactome after exercise. This approach will allow us to identify key proteins that dynamically interact with the PHGDH HHTH domain across different cell types and subcellular compartments, thus revealing the specific regulatory mechanisms involved during physical activity.

Second, we will construct organoid models. Specifically, we will introduce a PHGDH-luc reporter gene into late-onset Alzheimer’s disease brain organoids (LOAD-BOs) to simulate and study the regulatory effects of exercise on PHGDH. This platform will be used to screen pharmacological compounds that can mimic the beneficial effects of exercise. Such screening will not only validate the generalizability of our mechanistic findings but also identify promising therapeutic targets and lead compounds for future drug development. We are confident that the synergistic combination of these two strategies will open new avenues for elucidating the molecular mechanisms by which exercise regulates PHGDH.

## 7. Discussion

### 7.1. The Mechanistic Duality of PHGDH: From Specific Molecular Targets to General Longevity Pathways

The findings of this review underscore a pivotal shift in our understanding of neurodegenerative disease management, moving from symptomatic treatment to targeting fundamental metabolic and longevity pathways. At the specific molecular level, the most compelling data presented herein concern the spatial reprogramming of phosphoglycerate dehydrogenase (PHGDH). We observed that under the pathological stress of Alzheimer’s disease (AD), PHGDH undergoes a profound functional switch. While canonically understood as a cytoplasmic rate-limiting enzyme for serine synthesis—essential for synaptic plasticity and neuronal survival—PHGDH translocates to the nucleus in astrocytes under conditions of glucose deprivation or stress [4,7]. In this nuclear compartment, utilizing its helix–helix–turn–helix (HHTH) domain, PHGDH functions as a pathogenic transcription factor that independently drives the expression of pro-inflammatory genes such as IKKα and HMGB1 [4,10]. This specific mechanism elucidates why previous interventions targeting general metabolism may have failed; the pathology is driven not merely by the presence of the enzyme, but by its aberrant spatial localization and non-canonical gene-regulatory activity.

Focusing on the central nervous system, the review highlights that physical exercise exerts its neuroprotective effects precisely by counteracting this spatial dysregulation. The specific mechanism involves the exercise-induced myokine irisin, which crosses the blood–brain barrier and activates the AMPK/PGC-1α axis [14,15]. This signaling cascade appears to be critical for maintaining PHGDH in its cytoplasmic, metabolic role, thereby preventing its nuclear translocation and the subsequent suppression of autophagy and acceleration of amyloid-beta (Aβ) deposition [4,15]. This represents a highly specific molecular intervention where lifestyle factors (exercise) directly modulate the subcellular dynamics of a key metabolic enzyme to restore homeostasis.

Expanding to the systemic level, the “liver–brain–immune axis” introduces a broader dimension to the concept of longevity interventions. The reviewed data demonstrate that in aged or metabolically stressed murine models, PHGDH is upregulated specifically in hepatic Kupffer cells, promoting serine flux toward IL-1β biosynthesis via the NLRP3 inflammasome [15]. In this pathway, serine-derived metabolites fuel mitochondrial respiration, enhancing ROS generation and caspase-1 activation, which cleaves pro-IL-1β into its active form [15]. While studies using the PHGDH inhibitor NCT-503 in murine models have reported reductions in IL-1β exceeding 40%, the specific dosing regimens, duration of treatment, and whether these effects represent direct inhibition of Kupffer cell PHGDH or indirect modulation through other cell types require further clarification [12,15].

Regarding the connection between peripheral inflammation and central nervous system pathology, current evidence suggests that in neurodegenerative contexts where BBB integrity is compromised, elevated circulating IL-1β may contribute to microglial activation and exacerbation of Aβ/tau pathology [15]. However, the precise mechanisms by which IL-1β crosses the BBB in AD, the relative contribution of peripheral versus central IL-1β production, and whether IL-1β acts primarily through direct signaling or as part of a broader inflammatory cascade remain areas of active investigation [15]. Future research should employ cell-type-specific PHGDH knockout models and detailed pharmacokinetic studies to elucidate these mechanisms more precisely [12].

Furthermore, the discussion of pharmacological mimetics, such as the BBB-penetrant inhibitor NCT-503, provides a critical contrast to exercise interventions. While NCT-503 effectively disrupts the DNA-binding capacity of PHGDH and reduces Aβ plaques [4,12], it lacks the pleiotropic systemic benefits of exercise. However, the synergistic potential discussed—where exercise may enhance the bioavailability of such drugs or vice versa—points toward a future where “exercise–pharmacotherapy” becomes a standard for precision medicine in aging populations [4,12]. This aligns with the general objective of longevity research: to extend not just lifespan, but healthspan, by integrating biological, behavioral, and pharmacological strategies.

### 7.2. Limitations of the Review and Included Studies

While this narrative review synthesizes cutting-edge research, it is subject to specific limitations that must be acknowledged. Firstly, as a narrative review, the methodology employed relies on a non-systematic selection of literature to construct a logical argument regarding PHGDH and exercise. Although comprehensive searches were conducted, the lack of a quantitative meta-analysis means that the weight of evidence for specific mechanisms, such as the irisin-AMPK-PHGDH axis, is based on qualitative interpretation rather than statistical aggregation. This introduces the potential for selection bias, where studies supporting the proposed hypothesis may be emphasized over contradictory data.

Regarding the studies included in this review, significant limitations exist, particularly regarding the translational gap between preclinical models and human physiology. The majority of the mechanistic evidence detailing PHGDH nuclear translocation and its specific role as a transcription factor is derived from transgenic animal models (e.g., 3xTg-AD mice) and in vitro systems (e.g., brain organoids) [4,13]. While these models provide high-resolution insight into molecular pathways, they may not fully replicate the complex, multifactorial etiology of sporadic AD in humans. Additionally, the exercise protocols applied in animal studies often involve forced treadmill running or voluntary wheel running, which does not perfectly mirror the intensity, duration, or psychological context of human physical activity interventions. Consequently, while the mechanistic pathways identified in rodents are highly suggestive, their direct applicability to human patients remains to be fully validated.

Furthermore, the clinical data cited regarding the epidemiological benefits of exercise and the correlation of PHGDH with cognitive decline are largely observational. While these studies establish a strong association, they cannot definitively prove causality. For instance, the reduction in IL-1β and improved cognitive outcomes observed with exercise may be influenced by confounding variables such as diet, social interaction, or general health status, which are difficult to isolate in human cohorts.

### 7.3. Future Directions and Clinical/Research Impact

The insights gained from this review chart several critical directions for future research and hold profound implications for both clinical practice and scientific inquiry.

Future Directions: Future research should prioritize understanding the nuanced relationship between exercise, PHGDH inhibition, and neuroinflammation. While current evidence from murine models using PHGDH inhibitors like NCT-503 shows promising reductions in IL-1β, several critical questions remain unanswered. Studies are needed to determine: (1) the optimal dosing and treatment duration for PHGDH inhibitors; (2) whether these effects are direct consequences of Kupffer cell PHGDH inhibition or involve indirect modulation through other cell types; (3) the precise mechanisms by which peripheral IL-1β influences central nervous system pathology in AD; and (4) how exercise might modulate these pathways in a cell-type-specific manner. Addressing these questions will require innovative approaches, including cell-type-specific PHGDH knockout models, detailed pharmacokinetic/pharmacodynamic analyses, and advanced techniques to track the movement of inflammatory mediators across the BBB.

Clinical Impact: At the clinical level, this review advocates for a paradigm shift in the management of neurodegenerative diseases and the promotion of longevity. By reframing exercise as a precision intervention that modulates specific molecular targets (PHGDH), clinicians can move beyond generic lifestyle advice to prescribing tailored physical activity programs designed to alter specific metabolic pathways. The identification of PHGDH as a dual-function protein suggests that “one-size-fits-all” nutritional interventions, such as serine supplementation, may be ineffective or harmful in AD patients with elevated PHGDH expression [4]. Consequently, clinical protocols must evolve to include metabolic and genetic profiling to personalize dietary and therapeutic recommendations.

Research Impact: In the broader research landscape, this review contributes to the emerging field of “moonlighting proteins” in metabolism and aging. By highlighting the role of a metabolic enzyme as a transcription factor, it challenges the traditional boundaries of signal transduction and encourages researchers to investigate non-canonical functions of other metabolic hubs. The concept of “spatial reprogramming” offers a new lens through which to view aging and neurodegeneration, suggesting that restoring the correct cellular architecture of proteins is as important as modulating their abundance. This perspective is likely to stimulate interdisciplinary research combining structural biology, neurology, and exercise physiology to unlock novel therapeutic targets for longevity.

## 8. Conclusions

This narrative review successfully achieved its objectives by elucidating the complex role of PHGDH in neurodegeneration and the beneficial regulatory mechanisms of exercise. Specifically, we deciphered the dual role of PHGDH in astrocytes, distinguishing its canonical metabolic function, which supports synaptic plasticity, from its pathological function as a nuclear transcription factor driving IKKα- and HMGB1-mediated neuroinflammation and Aβ deposition. We determined the mechanisms by which exercise reprograms PHGDH function, highlighting central effects in which exercise-induced irisin activates AMPK/PGC-1α to suppress nuclear translocation, and peripheral effects in which exercise suppresses PHGDH in hepatic macrophages to reduce systemic IL-1β release. Furthermore, we outlined the translational potential of PHGDH-targeted interventions, proposing a framework for PET probes of the PHGDH HHTH domain and synergistic exercise–pharmacotherapy combinations that may leverage exercise-induced adaptations in blood–brain barrier function and transport mechanisms.

These findings have profound implications, bridging a critical gap between metabolic regulation and neuroimmunology and offering a robust alternative to the therapeutic stalemate in Alzheimer’s disease. At the clinical level, this review establishes a foundation for “precision exercise medicine,” enabling early diagnosis and patient stratification using biomarkers of PHGDH subcellular dynamics. Practical applications include the development of exercise-mimetic drugs, such as NCT-503, and the design of personalized exercise prescriptions tailored to maximize PHGDH regulation. By shifting the focus from general neuroprotection to precise metabolic interventions targeting PHGDH spatial dynamics, this review provides an actionable roadmap for improving AD patient outcomes and advancing effective healthy aging strategies.

## Figures and Tables

**Table 1:** Characteristics of key studies included in the review.

Citation	Country	Objective	Design	Participants/Model	Intervention	Results	Main Conclusion
[2]	Not specified	To investigate the non-canonical transcriptional role of PHGDH and the therapeutic effect of its inhibition.	Experimental Study	APP-KI mice; 3xTg-AD mice; Human brain organoids	NCT-503 (PHGDH inhibitor); Genetic overexpression	NCT-503 reduced hippocampal Aβ plaques by 44–62% and rescued spatial memory deficits. Enzyme-dead mutants retained pathogenic function.	PHGDH functions as a pathogenic transcription factor independent of its enzymatic activity; targeting its DNA-binding capacity is a viable therapeutic strategy.
[4]	Not specified	To investigate dynamic changes of PHGDH expression during AD pathological and symptomatic progression, and validate its biomarker potential.	Observational Validation Study	3xTg-AD mice; PS19 tauopathy mice; Multi-cohort human AD samples (Mayo, ROSMAP, Amsterdam, Baltimore cohorts)	Retrospective reanalysis of RNA-seq, single-nucleus transcriptome, proteomic datasets; Immunohistochemical validation	1. PHGDH expression was progressively elevated in AD mouse models with pathology development. 2. Astrocytic PHGDH increased sequentially from no to early/late AD pathology in humans. 3. PHGDH levels negatively correlated with cognitive function (DRS score). 4. Corrected prior bias caused by postmortem delay (PMD) in PHGDH detection.	PHGDH expression increases with AD pathological and symptomatic progression, serving as a potential early diagnostic biomarker; oral serine supplementation for AD requires caution.
[10]	Not specified	To determine the mechanism of PHGDH nuclear translocation under metabolic stress.	Mechanistic Study	Cancer cell lines (SW1990, LM3); Nude mouse xenograft model; 92 human pancreatic cancer specimens	Glucose deprivation; Hypoxia; p38/AMPK/JNK inhibitors; NCT-503; PHGDH/c-Jun mutagenesis (S371A, S55A, R236E); PJ34	1. p38 phosphorylates PHGDH-Ser371 to drive nuclear translocation; AMPK phosphorylates PHGDH-Ser55 to enhance malate oxidation. 2. Nuclear PHGDH lowers nuclear NAD^+^ and inhibits PARP1-mediated c-Jun PARylation. 3. Nuclear PHGDH supports tumor growth under nutrient stress. 4. PHGDH-Ser371/Ser55 phosphorylation correlates with p38/AMPK activity in clinical tumors.	Nutrient stress controls PHGDH nuclear translocation and alternative activity via p38/AMPK dual phosphorylation. Nuclear PHGDH maintains tumor growth through the NAD^+^-PARP1-c-Jun axis independently of serine synthesis.
[11]	Not specified	To summarize the effects and mechanisms of exercise and exercise mimetics on adult hippocampal neurogenesis (AHN) and cognition in neurodegenerative diseases, and discuss translational prospects.	Narrative Review	AD/PD/HD transgenic mice; Neural stem cells; Human postmortem brain tissues; Clinical cohorts	Physical exercise (wheel running, treadmill, swimming); Exercise mimetics (irisin, BDNF, metformin, AICAR, miRNAs, SAM, clusterin); Fndc5 knockout; BDNF mutation	1. Exercise enhances AHN, synaptic plasticity and cognition via muscle–brain/liver–brain/gut-brain axes. 2. Exercise mimetics replicate neuroprotective effects by activating AMPK/PGC-1α and alleviating neuroinflammatio. 3. Impaired AHN is a core pathological feature of AD, PD and HD.	Exercise and exercise mimetics effectively boost hippocampal neurogenesis and counteract neurodegeneration; targeting exercise-mimetic molecules is a novel strategy for treating neurodegenerative diseases.
[12]	Not specified	To identify circulating blood factors that mediate exercise-induced improvements in hippocampal neurogenesis and cognition in aged mice, and to define the liver–brain axis mechanism.	Experimental Study	Hepatic macrophages (Kupffer cells); Mice	Exercise; Myokine exposure; GPLD1 modulation	Exercise suppressed GPLD1 in Kupffer cells, reducing systemic IL-1β release and neuroinflammation.	Exercise modulates peripheral immunity to protect the brain by suppressing GPLD1 activity in the liver.

## Data Availability

No new data were created or analyzed in this study.

## Abbreviation

3-PG	3-phosphoglycerate
3-PHP	3-phosphohydroxypyruvate
3xTg-AD	Triple-transgenic Alzheimer’s disease model
Aβ	Amyloid beta
AD	Alzheimer’s disease
AE	Aerobic exercise
AMPK	AMP-activated protein kinase
APP-KI	Amyloid precursor protein knock-in
ATPIF1	ATPase inhibitory factor 1
AXIN	Axis inhibition protein
BACE1	Beta-site amyloid precursor protein cleaving enzyme 1
BBB	Blood–brain barrier
BDNF	Brain-derived neurotrophic factor
BOs	Brain organoids
CNS	Central nervous system
FNDC5	Fibronectin type III domain-containing protein 5
Gpld1	Glycosylphosphatidylinositol specific phospholipase D1
HHTH	Helix–helix–turn–helix
HIPK2	Homeodomain-interacting protein kinase 2
HMGB1	High mobility group box 1
IKKα	Inhibitor of nuclear factor kappa-B kinase alpha
IL-1β	Interleukin-1 beta
LOAD	Late-onset Alzheimer’s disease
LTP	Long-term potentiation
mTOR	Mechanistic target of rapamycin
NAD^+^/NADH	Nicotinamide adenine dinucleotide (oxidized/reduced)
NF-κB	Nuclear factor kappa-B
NCT-503	PHGDH inhibitor
NLRP3	NLR family pyrin domain containing 3
NMDA	N-methyl-D-aspartate
p38 MAPK	p38 mitogen-activated protein kinase
PET	Positron emission tomography
PGC-1α	Peroxisome proliferator-activated receptor gamma coactivator 1-alpha
PHGDH	Phosphoglycerate dehydrogenase
PK/PD	Pharmacokinetic/pharmacodynamic
ROS	Reactive oxygen species
sAD	Sporadic Alzheimer’s disease
SAM	S-adenosylmethionine
TREM2	Triggering receptor expressed on myeloid cells 2

## References

[1] You Y., Shou X., Zhang X., Fan S., Chai R., Xue W., Hu Y., He Q. (2022). Psycho-Cardiological Disease: A Bibliometric Review From 2001 to 2021. Front. Cardiovasc. Med..

[2] Chen J., Hadi F., Wen X., Zhao W., Xu M., Xue S., Lin P., Calandrelli R., Richard J.L.C., Song Z. (2025). Transcriptional regulation by PHGDH drives amyloid pathology in Alzheimer’s disease. Cell.

[3] Wu Y.Q., Zhang C.S., Xiong J., Cai D.Q., Wang C.Z., Wang Y., Liu Y.H., Wang Y., Li Y., Wu J. (2023). Low glucose metabolite 3-phosphoglycerate switches PHGDH from serine synthesis to p53 activation to control cell fate. Cell Res..

[4] Chen X., Calandrelli R., Girardini J., Yan Z., Tan Z., Xu X., Hiniker A., Zhong S. (2022). PHGDH expression increases with progression of Alzheimer’s disease pathology and symptoms. Cell Metab..

[5] Hu F., Peng J., Wang W., Shen L., Jia M. (2025). Comparing the impact of various exercise modalities on old adults with Alzheimer’s disease: A Bayesian network meta-analysis. Complement. Ther. Clin. Pract..

[6] De la Rosa A., Olaso-Gonzalez G., Arc-Chagnaud C., Millan F., Salvador-Pascual A., García-Lucerga C., Blasco-Lafarga C., Garcia-Dominguez E., Carretero A., Correas A.G. (2020). Physical exercise in the prevention and treatment of Alzheimer’s disease. J. Sport Health Sci..

[7] Raji C.A., Meysami S., Hashemi S., Garg S., Akbari N., Ahmed G., Chodakiewitz Y.G., Nguyen T.D., Niotis K., Merrill D.A. (2024). Exercise-Related Physical Activity Relates to Brain Volumes in 10,125 Individuals. J. Alzheimers Dis..

[8] da Rocha J.F., Lance M.L., Luo R., Schlachter P., Moreira L., Iqbal M.A., Kuhn P., Gardner R.S., Valaris S., Islam M.R. (2025). Protective exercise responses in the dentate gyrus of Alzheimer’s disease mouse model revealed with single-nucleus RNA-sequencing. Nat. Neurosci..

[9] Madhu L.N., Somayaji Y., Shetty A.K. (2022). Promise of irisin to attenuate cognitive dysfunction in aging and Alzheimer’s disease. Ageing Res. Rev..

[10] Ma C., Zheng K., Jiang K., Zhao Q., Sha N., Wang W., Yan M., Chen T., Zhao Y., Jiang Y. (2021). The alternative activity of nuclear PHGDH contributes to tumour growth under nutrient stress. Nat. Metab..

[11] Zhao R. (2024). Can exercise benefits be harnessed with drugs? A new way to combat neurodegenerative diseases by boosting neurogenesis. Transl. Neurodegener..

[12] Horowitz A.M., Fan X., Bieri G., Smith L.K., Sanchez-Diaz C.I., Schroer A.B., Gontier G., Casaletto K.B., Kramer J.H., Williams K.E. (2020). Blood factors transfer beneficial effects of exercise on neurogenesis and cognition to the aged brain. Science.

[13] Shan X., Hu P., Ni L., Shen L., Zhang Y., Ji Z., Cui Y., Guo M., Wang H., Ran L. (2022). Serine metabolism orchestrates macrophage polarization by regulating the IGF1-p38 axis. Cell Mol. Immunol..

[14] Cai Z., Li W., Hager S., Wilson J.L., Afjehi-Sadat L., Heiss E.H., Weichhart T., Heffeter P., Weckwerth W. (2024). Targeting PHGDH reverses the immunosuppressive phenotype of tumor-associated macrophages through α-ketoglutarate and mTORC1 signaling. Cell Mol. Immunol..

[15] Kong J., Xie Y., Fan R., Wang Q., Luo Y., Dong P. (2025). Exercise orchestrates systemic metabolic and neuroimmune homeostasis via the brain-muscle-liver axis to slow down aging and neurodegeneration: A narrative review. Eur. J. Med. Res..

[16] Wu X., Li C., Ke C., Huang C., Pan B., Wan C. (2024). The activation of AMPK/PGC-1α/GLUT4 signaling pathway through early exercise improves mitochondrial function and mitigates ischemic brain damage. Neuroreport.

[17] Bono F., Tomasoni Z., Mutti V., Sbrini G., Kumar R., Longhena F., Fiorentini C., Missale C. (2023). G Protein-Dependent Activation of the PKA-Erk1/2 Pathway by the Striatal Dopamine D1/D3 Receptor Heteromer Involves Beta-Arrestin and the Tyrosine Phosphatase Shp-2. Biomolecules.

[18] Hynes T.J., Chernoff C.S., Hrelja K.M., Tse M.T.L., Avramidis D.K., Lysenko-Martin M.R., Calderhead L., Kaur S., Floresco S.B., Winstanley C.A. (2024). Win-Paired Cues Modulate the Effect of Dopamine Neuron Sensitization on Decision Making and Cocaine Self-administration: Divergent Effects Across Sex. Biol. Psychiatry.

[19] Zhou F., Wei L., Wang Y., Chen W. (2024). Aerobic exercise modulates the striatal Erk/MAPK signaling pathway and improves LID in a mouse model of Parkinson’s disease. Brain Res. Bull..

